# Recent Developments on the Synthesis and Bioactivity of Ilamycins/Rufomycins and Cyclomarins, Marine Cyclopeptides That Demonstrate Anti-Malaria and Anti-Tuberculosis Activity

**DOI:** 10.3390/md19080446

**Published:** 2021-08-03

**Authors:** Uli Kazmaier, Lukas Junk

**Affiliations:** 1 Organic Chemistry, Saarland University, Campus Building C4.2, 66123 Saarbrücken, Germany; l.junk@mx.uni-saarland.de; 2 Helmholtz Institute for Pharmaceutical Research Saarland (HIPS)—Helmholtz Centre for Infection Research (HZI), Campus Building E8 1, 66123 Saarbrücken, Germany

**Keywords:** ilamycins, rufomycins, cyclomarins, tuberculosis, malaria, cyclopeptides, biosynthesis, total synthesis, natural products

## Abstract

Ilamycins/rufomycins and cyclomarins are marine cycloheptapeptides containing unusual amino acids. Produced by *Streptomyces* sp., these compounds show potent activity against a range of mycobacteria, including multidrug-resistant strains of *Mycobacterium tuberculosis*. The cyclomarins are also very potent inhibitors of *Plasmodium falciparum*. Biosynthetically the cyclopeptides are obtained via a heptamodular nonribosomal peptide synthetase (NRPS) that directly incorporates some of the nonproteinogenic amino acids. A wide range of derivatives can be obtained by fermentation, while bioengineering also allows the mutasynthesis of derivatives, especially cyclomarins. Other derivatives are accessible by semisynthesis or total syntheses, reported for both natural product classes. The anti-tuberculosis (anti-TB) activity results from the binding of the peptides to the *N*-terminal domain (NTD) of the bacterial protease-associated unfoldase ClpC1, causing cell death by the uncontrolled proteolytic activity of this enzyme. Diadenosine triphosphate hydrolase (PfAp3Aase) was found to be the active target of the cyclomarins in *Plasmodia*. SAR studies with natural and synthetic derivatives on ilamycins/rufomycins and cyclomarins indicate which parts of the molecules can be simplified or otherwise modified without losing activity for either target. This review examines all aspects of the research conducted in the syntheses of these interesting cyclopeptides.

## 1. Introduction

Marine organisms produce a wealth of natural products, creating a universe of fascinating new chemical structures [1,2]. These natural products are often the result of an evolutionary process providing competitive advantages to their producers in their natural environments. Therefore, many of these natural products have notable biological activities, making them good candidates for drug development [3,4,5], including against infectious diseases such as malaria and tuberculosis.

Malaria is one of the most common tropical diseases, with more than 200 million infections and 600,000 deaths annually worldwide [6], mainly in the poorest population. Tuberculosis (TB) is also common: in 2019, approximately 10 million people fell ill with the disease and 1.5 million died [7]. In addition, in 2018, 500,000 people demonstrated resistance to rifampicin, the most effective first-line drug, 80% of whom suffer from multidrug-resistant tuberculosis (MDR-TB). The development of antibiotic resistance is widespread, and these multi-resistant pathogens are a particularly serious problem. Therefore, new drugs are required [8]. Most first- and second-line drugs were discovered or developed between 1940 and 1980, often with a similar mode of action, facilitating the development of resistance [9,10]. Modern drugs should therefore work via new modes of action against not only the MDR-TB strains but also the largely drug-resistant tuberculosis (XDR-TB) strains, which are now almost resistant to all drugs. In addition, any new drug should effectively destroy dormant bacteria, beneficial for short-term therapies, and be nontoxic (because of the generally long treatment times required). Natural products are excellent candidates for developing anti-TB drugs, and more than 60% of drugs under current development are natural products or derived from natural products [11,12,13].

## 2. Discovery of Anti-Tubercular Cycloheptapeptides

### 2.1. Discovery of the Ilamycins/Rufomycins

In 1962, two independent research groups investigated marine *Streptomycetes* from soil samples found on Japanese islands. Takita et al. observed that the culture filtrate of a new strain, *Streptomyces insulates* (A-165-Z1), later renamed *Streptomyces islandicus*, inhibited the growth of *Mycobacterium* 607 and *Mycobacterium phlei*. They isolated two antibiotics and named them ilamycin A and B (IlaA and IlaB) [14,15].

In addition, at this time Shibata et al. isolated two new antibiotics, rufomycin I and II (Ruf I and Ruf II), from the newly discovered *Streptomyces atratus* (46408), found to be especially active against acid-fast bacteria [16,17]. The compounds were also active against *Mycobacterium tuberculosis* and *Mycobacterium smegmatis* but almost inactive against most other bacteria, fungi, and yeasts. Subsequent research indicated that these two antibiotics possess very similar chemical structures [18,19,20,21,22,23]. 

**Figure 1:**
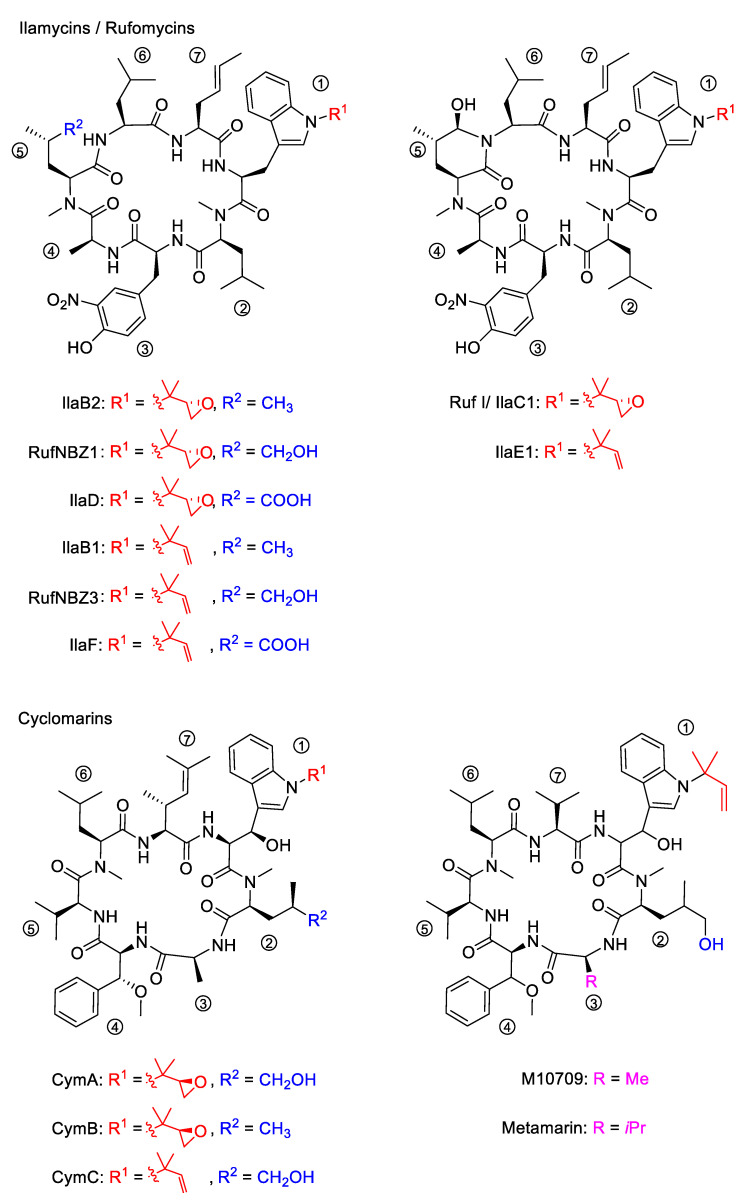
Selected ilamycins/rufomycins and cyclomarins.

The structures of ilamycins/rufomycins (Figure 1) are unusual, as these cyclic heptapeptides contain a series of atypical amino acids. Most prominent is the *N*-prenylated tryptophan ①, which can also be found in the epoxidized form [24,25]. At the *N*-terminus of the tryptophan, a γ,δ-unsaturated amino acid is incorporated ⑦ [26]. Common to all derivatives is a unique 3-nitrotyrosine ③, a building block not found in any other natural product. The greatest variability is observed in the leucine building block ⑤, which can be oxidized to different oxidation levels at a terminal methyl group. In its original description, ilamycin was proposed to contain an aldehyde functionality [23], but structural elucidation by NMR and X-ray crystallography showed that the aldehyde functionality undergoes cyclization with the nearby amide bond [27,28,29]. Very recently, a wide range of new ilamycins/rufomycins were described, differing mainly in the combination of different amino acid oxidation levels ⑤ and the *N*-prenyl substituent of ① (alkene, epoxide, diol) [27,28,29].

### 2.2. Discovery of the Cyclomarins

In 1999, the research groups of Fenical and Clardy reported the isolation of three new anti-inflammatory cyclic peptides from extracts of a *Streptomyces* sp. collected in Mission Bay, California [30]. These secondary metabolites from the strain CNB-982, called cyclomarins (Cym) A−C, are structurally related to the rufomycins. Very similar amino acid building blocks are incorporated, although in a different sequence. As in the rufomycins, an *N*-prenylated tryptophan ①(CymC) is a notable building block that can also be epoxidized (CymA). However, in contrast to the rufomycins, in the cyclomarin series, the tryptophan units are β-hydroxylated. At the *N*-terminus of the tryptophan, a γ,δ-unsaturated amino acid is incorporated, not a linear one as found in the rufomycins, but one that is branched and dimethylated ⑦. One of the leucines is also oxidized at the δ position ②, at least in CymA and C, but at another position, as in the rufomycins. Most obvious is the replacement of the unique nitrotyrosine by another aromatic amino acid, *syn*-β-methoxyphenylalanine ④.

In 2010, the group of Mikami described the extraction of a new cyclomarin derivative M10709 from clinically isolated *Streptomyces* sp. IFM 10,709 [31]. Although not all stereogenic centers were determined properly, results revealed the compound was different from cyclomarin C only by the replacement of the unsaturated amino acid ⑦ by valine. The same structural motif was also found in a recently isolated metamarine in which a valine at position ③ replaced an alanine. The discovery of metamarine resulted from a larger soil metagenome project undertaken to discover rufomycin/cyclomarin-like antibiotics [32].

## 3. Biosynthesis of Anti-Tubercular Cycloheptapeptides

Although the ilamycins/rufomycins were originally described in the early 1960s, biosynthetic studies of these unusual cyclopeptides were reported only recently [27,33]. The first investigations of cyclomarins were conducted in 2008 by the Moore group [34,35], while Brady et al. reported their results on metamarine biosynthesis in 2021 [32].

**Figure 2:**
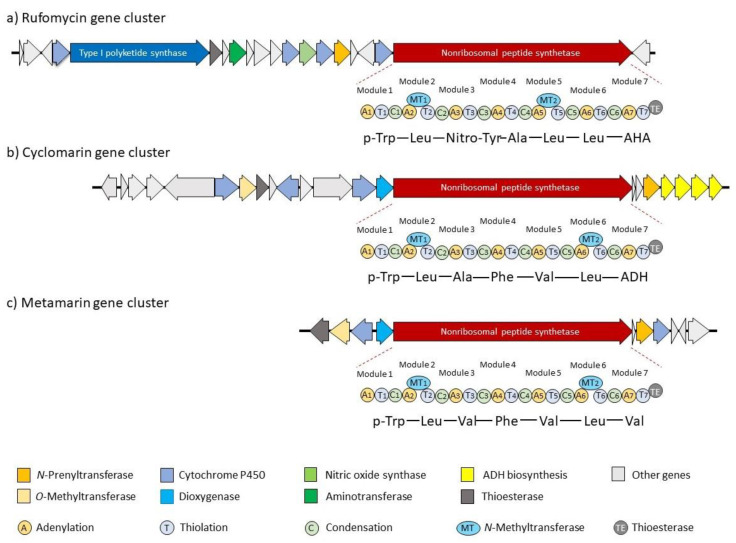
Biosynthesis of rufomycins and (modified) cyclomarins; (**a**) Rufomycin gene cluster, (**b**) Cyclomarin gene cluster, (**c**) Metamarin gene cluster.

Although there are significant differences in the structures of these cycloheptapeptides, they also have structural similarities and common biosynthetic features (Figure 2). A key feature of the biosynthetic gene cluster is a gene encoding a heptamodular nonribosomal peptide synthetase (NRPS). This NRPS is surrounded by a set of open reading frames (ORF) that encodes tailoring enzymes (P450, oxygenases) and provides the required building blocks for peptide synthesis. They contain the unusual amino acids *N*-prenyl-tryptophan (p-Trp), 3-nitrotyrosine (Nitro-Tyr, ilamycins), as well as the unsaturated amino acids (2*S*,4*E*)-2-amino-4-hexenoic acid (AHA, ilamycins) and (2*S*,3*R*)-2-amino-3,5-dimethylhexenoic acid (ADH, cyclomarins). These building blocks are directly incorporated into the NRPS, while oxidative tailoring enzymes encoded in the ORFs modify the protein-bound peptide during synthesis. All biosyntheses start with the p-Trp ① and end with a cyclization between ① and the C-terminal amino acids ⑦.

### 3.1. Biosynthesis of the Ilamycins/Rufomycins

A total of 20 genes are involved in the biosynthesis of these cyclopeptides (Figure 2a) [27,36]. Besides the gene encoding the NRPS, a second large gene encodes a type I polyketide synthase (PKS), which is involved in AHA synthesis. (*E*)-4-Hexenic acid is obtained from AcCoA, and is further hydroxylated, oxidized (P450), and transaminated to AHA [33]. An *N*-prenyltransferase is responsible for the generation of the first amino acid of the sequence from tryptophan. The unusual 3-nitrotyrosine is obtained by P450-catalyzed nitrosation of tyrosine, and the NO required is provided by a nitric acid synthase from arginine [33]. Additional P450 cytochrome controls the final oxidation steps to create the different ilamycin family members [27].

### 3.2. Biosynthesis of Cyclomarins

**Figure 3:**
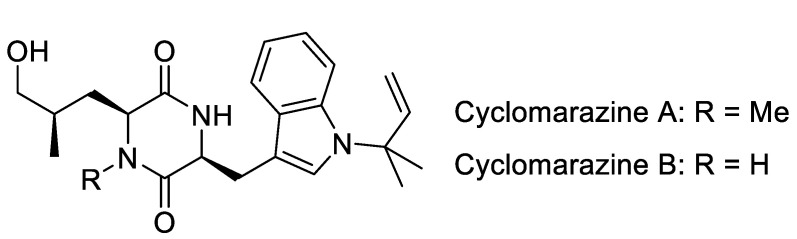
Cyclomarazines.

Although the cyclomarins are structurally related to the ilamycins, they are significantly different, at least from a biosynthetic point of view (Figure 2b) [34,35]. In contrast to the ilamycins, the linear unsaturated amino acid AHA is replaced by the dimethylated derivative ADH, which has a completely different biosynthetic origin. While AHA is obtained by a classical polyketide synthase, ADH is formed from valine and pyruvate [35]. Of the 23 ORFs of the biosynthetic gene cluster, several genes are responsible for ADH synthesis. Another significant difference is the β-OH functionality of the prenylated tryptophan (p-Trp), which is found in all cyclomarins but not in the ilamycins and rufomycins. Its introduction is catalyzed by a tryptophan-β-hydroxylase, a dioxygenase, and occurs on the protein-bound peptide in the early stages of the biosynthesis. In contrast, epoxidation of the *N*-prenyl side chain by a P450 epoxidase occurs post-NRPS assembly. Gene inactivation of the prenyltransferase eliminated the production of cyclomarin, indicating that p-Trp is the initiator of the peptide assembly line and that the unmodified p-Trp is not a good substrate for the first acceptor domain [34]. The β-hydroxylation must occur during the first two steps of the peptide synthesis, since inactivation of the corresponding gene does not result in the formation of a desoxycyclomarin but the formation of a dioxopiperazine called cyclomarazine (Figure 3), containing only the first two amino acids of the biosynthesis.

CymA and CymB were isolated from the *Streptomyces* sp. CNB-982, together with CymD that was missing the *N*-methyl group on the δ-hydroxyleucine ②. It is clear that δ-hydroxylation by P450 leucine hydroxylase occurs faster than *N*-methylation, and tryptophan hydroxylation is essential for prolonging the peptide chain. If this hydroxylation does not occur properly, the incompletely processed dipeptide is cleaved from the NRPS due to ineffective processing by the next module. This reaction is a unique example in which a megasynthetase can produce two different natural products of different sizes simply by triggering the level of β-oxidation on the priming Trp unit. Notably, cyclomarazines do not show significant biological activity.

The second major way in which cyclomarins differ from ilamycins is the incorporation of β-methoxyphenylalanine (MeOPhe) instead of nitrotyrosine. This amino acid is obtained from a peptide-bound Phe via a P450-catalyzed β-hydroxylation followed by *O*-methylation [35].

During their soil metagenome project, Brady et al. also investigated the biosynthesis of metamarin. In this cyclomarin derivative, the unsaturated amino acid is replaced by valine (Figure 2c) [32]. Another valine is incorporated instead of alanine ③. Therefore, it is not surprising that the gene cluster is comparable to the cyclomarin cluster, and only the genes responsible for ADH biosynthesis are missing.

## 4. Total Syntheses of Marine Cycloheptapeptides

The interesting biological properties and unusual building blocks of marine cycloheptapeptides sparked the interest of synthetic chemists, and the syntheses of several different amino acids and fragments have been reported in a recent review [37]. Therefore, they will not be discussed in detail here, and the focus will be on the total syntheses of the natural products.

### 4.1. Total Synthesis of Ilamycins/Rufomycins

**Scheme 1:**
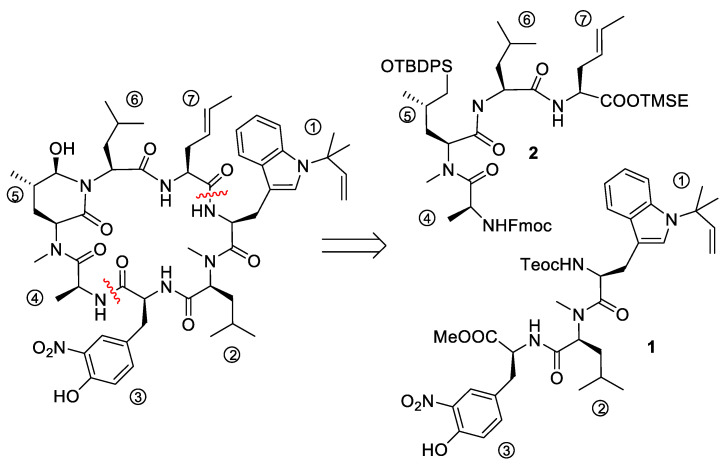
Retrosynthesis of ilamycins (Guo and Ye).

To date, only one synthetic route has been described for ilamycins E1 and F by Guo and Ye et al. [38]. In their highly convergent strategy, the ilamycins were synthesized from two parts (**1** and **2**) that were linked between ① and ⑦ to the macrocyclic lactam (Scheme 1). The lower right tripeptide part **1** (①–③) was prepared in five steps from tryptophan, while the upper left tetrapeptide **2** (④–⑦) required 13 steps from glutamic acid. Final oxidation of the δ-hydroxyleucine ⑤ resulted in the described ilamycins.

**Scheme 2:**
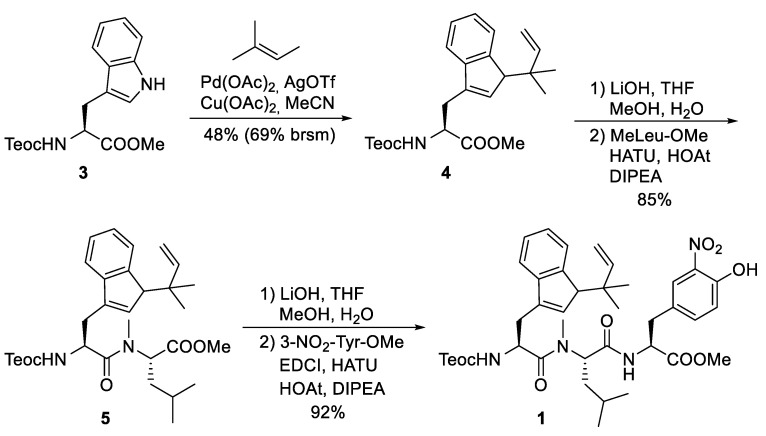
Synthesis of peptide fragment **1**.

The synthesis of peptide fragment **1** was rather straightforward (Scheme 2). 2-(Trimethylsilyl)ethoxycarbonyl (Teoc)-protected tryptophan methylester **3** was subjected to a Pd-catalyzed *N*-*tert*-prenylation according to a protocol developed by Baran et al. [39]. Saponification of the ester moiety of **4** and peptide coupling with *N*-methylated Leu-OMe produced dipeptide **5**, which was further elongated to tripeptide **1**.

**Scheme 3:**
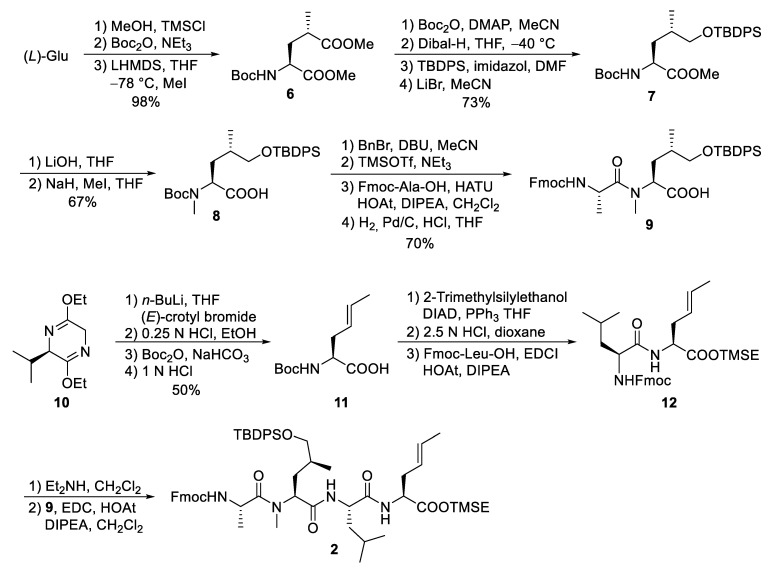
Synthesis of peptide fragment **2**.

For the larger fragment **2**, glutamic acid was converted into protected **8** (Scheme 3) according to a synthetic route developed during the synthesis of dysithiazolamide [40]. The glutamic acid was converted into the dimethyl ester and *N*-Boc protected before it was stereoselectively α-methylated at the sterically least-hindered ester moiety [41,42]. For the chemoselective reduction of the γ-ester **6**, a second *N*-Boc-protecting group was introduced, and the sterically least-hindered ester functionality was reduced with DIBAL-H. Silyl protection of the primary alcohol and subsequent mono-Boc deprotection yielded **7**. The methyl ester was saponified (to avoid α-methylation), and the Boc-amide was selectively *N*-methylated to **8** with NaH/MeI. The free carboxylic acid **8** was converted into the corresponding benzyl ester. TMSOTf/NEt_3_ was used for selective cleavage of the *N*-Boc-protecting group without affecting the OTBDPS group. The free amine could be coupled with Fmoc-protected alanine, and the *C*-terminal benzyl ester was cleaved by catalytic hydrogenation to provide the free acid **9**.

Schöllkopf auxiliary **10** [43] was subjected to stereoselective crotylation to generate the *C*-terminal unsaturated amino acid of **2** (Scheme 3). Subsequent auxiliary cleavage provided *N*-Boc-protected amino acid **11**, which was converted into the corresponding TMSE ester. Boc-deprotection and peptide coupling produced dipeptide **12**. Subsequent Fmoc deprotection and coupling with **9** generated the linear tetrapeptide **2**.

**Scheme 4:**
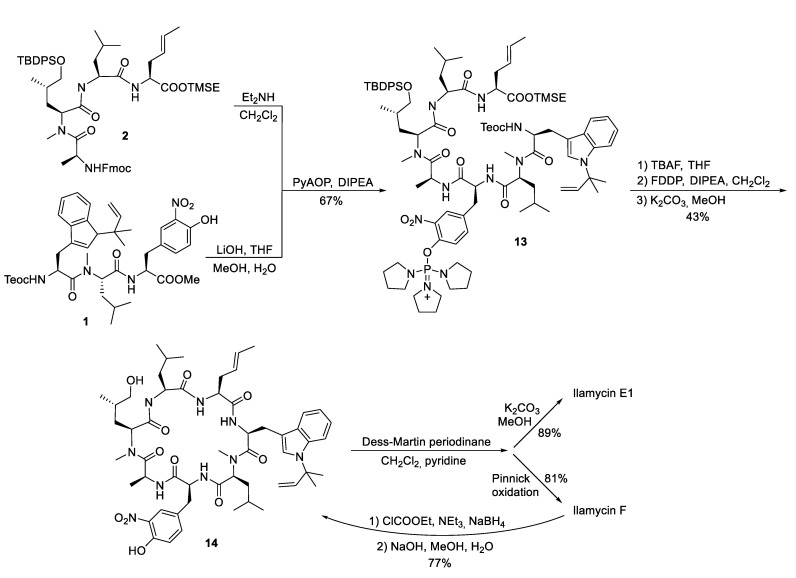
Synthesis of ilamycinE1 and F.

With the two major building blocks produced, ilamycin synthesis could proceed to the final step (Scheme 4). Mild saponification of the methylester **1** and coupling with Fmoc-deprotected **2** using (2-azabenzotriazol-1-yloxy)tripyrrolidinophosphonium hexafluorophosphate (PyAOP) [44] yielded **13** without significant epimerization. Global deprotection with TBAF resulted in the linear heptapeptide, which was subjected to macrolactamization. While many peptide coupling reagents have been investigated, the best results were obtained using pentafluorophenyl diphenylphosphinate (FDPP) [45]. As a side product in addition to the expected cyclopeptide **14**, the diphenylphosphinylated ester was formed, which could directly be converted into **14** by treatment with K_2_CO_3_ in methanol, providing an overall yield of 43% of the desired **14**. Finally, only the primary OH-functionality needed to be oxidized. Depending on the oxidation protocol, both ilamycin E1 and F could be obtained. Ilamycin E1 was obtained as a single stereoisomer. Notably, ilamycin F is also available on a gram scale via fermentation, but the derivative E1, which is approximately 100-fold more potent, is not. Therefore, the authors developed a protocol to convert ilamycin F into intermediate **14** by reducing the mixed anhydride, permitting an interconversion of ilamycin F into ilamycin E1 [38].

### 4.2. Total Synthesis of Cyclomarins

The amino acids present in the cyclomarins are slightly more complex than in the ilamycins, and various synthetic approaches have been undertaken to produce these building blocks and partial structures of cyclomarin [46]. These are covered in a recent review [37], and therefore only the routes applicable to the synthesis of cyclomarins and derivatives will be discussed here.

**Scheme 5:**
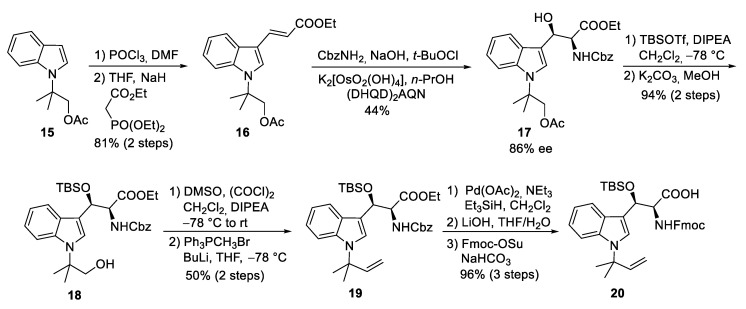
Synthesis of protected tryptophan **20** (building block ①).

The first synthesis of cyclomarin C was reported in 2004 by Yao and coworkers [47]. The unusual *tert*-prenylated β-hydroxy-tryptophan ① was obtained from indole derivative **15** (Scheme 5). This compound is available from indole via *N*-alkylation with ethyl-α-bromo-propionate, subsequent α-methylation of the ester, LAH-reduction, and acetylation [48]. Formylation and a subsequent Horner–Wadsworth–Emmons reaction yielded α,β-unsaturated ester **16**, which could be subjected to a Sharpless aminohydroxylation [49]. Moderate yield and enantioselectivity of the desired β-hydroxytryptophan derivative **17** was obtained. Unfortunately, no comment was made concerning the regioselectivity of the reaction. Silylation of the β-hydroxy group and selective transesterification of the acetate gave rise to primary alcohol **18**, which could be oxidized to the aldehyde and methenylated via Wittig reaction. Finally, the Cbz-protecting group from **19** was removed selectively without affecting the generated double bond. Furthermore, the free amine was Fmoc-protected after saponification of the ester. The use of the Fmoc- or Alloc-protecting group is essential for the synthesis of cyclomarins because other protecting groups, such as Boc, cannot be removed later on without side reactions, such as the elimination of the β-hydroxy functionality [50].

**Scheme 6:**
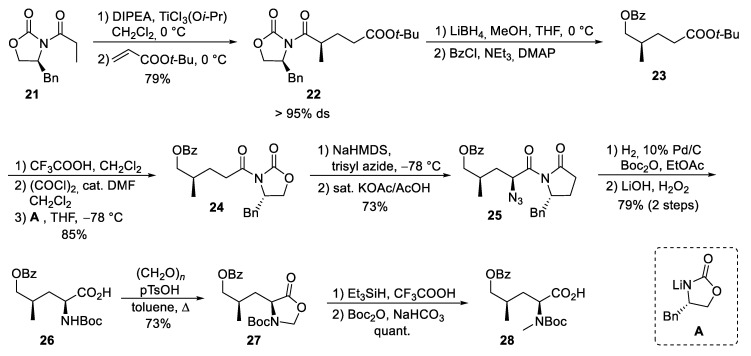
Synthesis of protected hydroxyleucine **28** (building block ②).

The synthesis of δ-hydroxyleucine building block ②, with the opposite configuration of the γ-methyl group than in amino acid ⑤ in the ilamycins, was obtained by classical asymmetric synthesis using chiral auxiliary chemistry (Scheme 6). According to Evans et al. [51], chiral oxazolidinone **21** was subjected as its titanium enolate in a Michael addition to *tert*-butyl acrylate to provide a good yield of **22** with high stereoselectivity. The imide was selectively reduced in the presence of the *tert*-butyl ester using LiBH_4_, and the resulting primary alcohol was *O*-benzoylated to **23**. Acidic cleavage of the *tert*-butyl ester, activation of the carboxylic acid, and reapplication of the Evans auxiliary **A** provided oxazolidinone **24**. Deprotonation of **24** and stereoselective azidation produced azide **25**. Catalytic hydrogenation in the presence of Boc_2_O resulted in the formation of the *N*-Boc-protected amino derivative, which was saponified to the corresponding amino acid **26**. The desired *N*-methyl group was introduced by conversion of **26** into the corresponding oxazolidinone **27**, which was reduced using triethylsilane in presence of trifluoroacetic acid. Subsequent cleavage of the Boc-protecting group required its reintroduction to **28**.

**Scheme 7:**
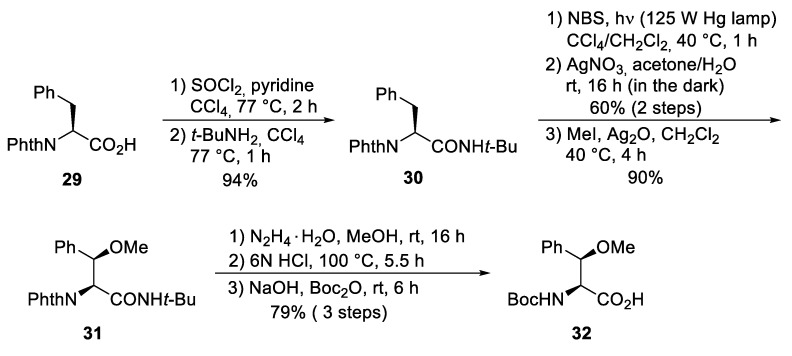
Synthesis of protected β-methoxy phenylalanine **32** (building block ④).

The unusual β-methoxyphenylalanine ④ was obtained from *N*-phthaloyl-protected phenylalanine **29**, which was converted into the corresponding *tert*-butylamide **30** (Scheme 7). Oxygen functionality was introduced into the β-position by subjecting **30** to a Wohl–Ziegler bromination, providing a 1:1 diastereomeric mixture of the desired β-bromo derivative [52]. According to Easton et al., treatment of the diastereomeric mixture with AgNO_3_ in aqueous acetone produced the desired (2*S*,3*R*)-β-hydroxyphenylalanine enantio- and diastereoselectively [53]. The stereochemical outcome can be explained by a preferred conformation of the benzylic carbenium ion in the substitution step. The best selectivities were obtained with the *tert*-butylamide. Subsequent *O*-methylation provided the methoxy derivative **31**, converted into the *N*-Boc-protected amino acid **32** under standard conditions.

**Scheme 8:**
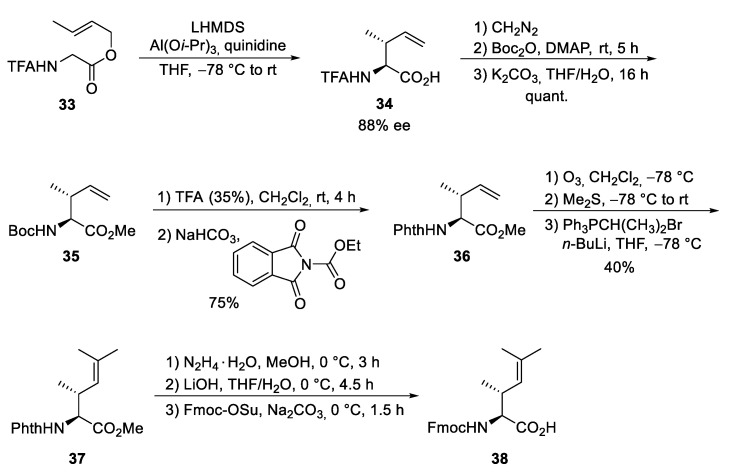
Synthesis of protected dehydroamino acid **38** (building block ⑦).

Finally, the unsaturated amino acid ⑦ was obtained via an asymmetric chelate enolate Claisen rearrangement, developed by Kazmaier et al. (Scheme 8) [54,55]. Trifluoroacetyl (TFA)-protected glycine crotyl ester **33** was deprotonated and converted into a chelated aluminum ester enolate, which in the presence of quinidine underwent a [3,3]-sigmatropic rearrangement to unsaturated amino acid **34** with good yield and enantioselectivity. Epimerization of the α-stereogenic center was avoided by first converting **34** into the Boc-protected ester **35** and then, in a second step, into the corresponding phthaloyl-protected derivative **36**. A direct epimerization-free conversion (**34** to **36**) was not possible. Ozonolysis of the double bond and subsequent Wittig reaction produced protected amino acid **37**, finally converted into the Fmoc-protected acid **38**.

**Scheme 9:**
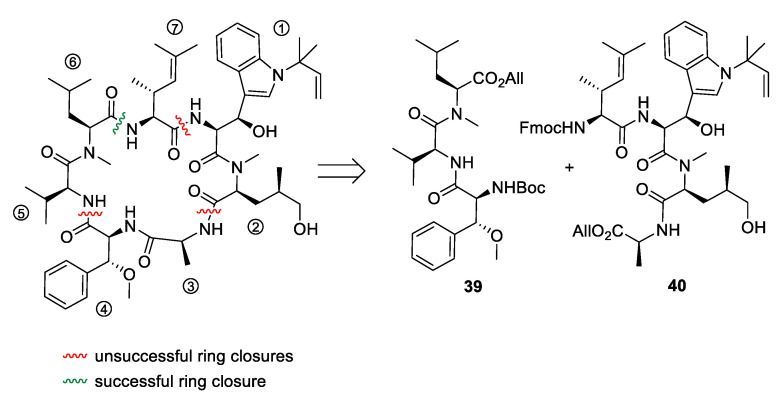
Cyclization attempts for cyclomarin C [56].

After the desired building blocks were created, the synthesis of cyclomarin C and especially the best position for macrocyclization was investigated (Scheme 9) [47,56]. An attempt to align the synthesis to the biosynthetic pathway and to cyclize the linear heptapeptide precursor between the unusual tryptophan ① and the unsaturated amino acid ⑦ failed. Although obtaining the linear peptide in a [3+3+1] peptide fragment coupling strategy was straightforward, the final deprotection and ring closure yielded only trace amounts of the desired product. The same was true for attempts to cyclize the linear heptapeptide between the methoxyphenylalanine ④ and valine ⑤. The trial to cyclize between the sterically less demanding hydroxyleucine ② and alanine ③ failed early in the synthesis stage. All attempts to prolong the ①,② dipeptide at the *N*-terminus failed. Under the basic conditions for Fmoc-deprotection, spontaneous cyclization to the corresponding diketopiperazine occurred, comparable to the previously discussed biosynthetic side reaction, which resulted in the formation of the cyclomarazines. The ultimately successful route was the cyclization between the unsaturated amino acid ⑦ and the *C*-terminal *N*-methylleucine ⑥. The linear heptapeptide was obtained via a [4+3]-coupling strategy. An allyl ester was used as the *C*-terminal protecting group to avoid the basic reaction conditions required for the saponification of the *C*-terminal ester, which caused problems in previous cyclization attempts.

**Scheme 10:**
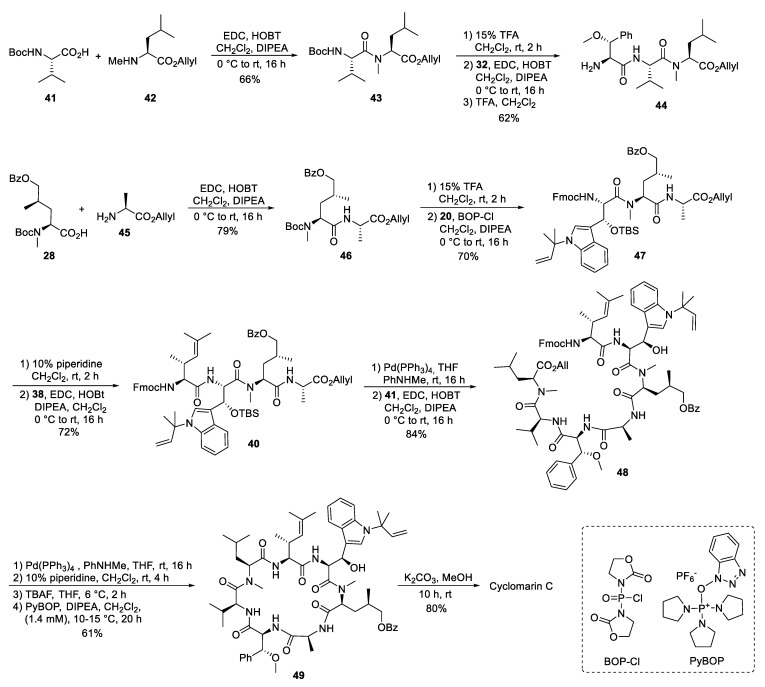
Synthesis of cyclomarin C.

The desired tri- and tetrapeptide **39** and **40** were synthesized using classical peptide coupling reactions and a combination of Boc- and Fmoc-protecting groups (Scheme 10). Because of the acid lability of β-hydroxytryptophan, Fmoc had to be used after incorporating this building block into the growing peptide chain. The synthesis of the peptide fragments was straightforward. An adequate yield of the tripeptide **39** was obtained from *N*-Boc-valine **41** and *N*-methylleucine allyl ester **42**. Boc-cleavage and coupling with methoxyphenylalanine **32** produced **39**, which was also *N*-deprotected to tripeptide **44**.

The synthesis of the tetrapeptide started with the coupling of protected δ-hydroxyleucine **28** with alanine allyl ester **45**. After *N*-deprotection, the Fmoc-protected tryptophan **20** was coupled using Bop-Cl/DIPEA [57]. Careful removal of the Fmoc-protecting group from **47** and EDC/HOBT-coupling with the unsaturated building block **38** provided tetrapeptide **40**. Finally, the *C*-terminal allyl ester was cleaved under mild Pd-catalyzed conditions, and the two peptide fragments were ready for the fragment coupling. An excellent yield of **48** was obtained using EDC/HOAt, which proved more suitable than HOBT. Subsequent deprotection of the *C*- and the *N*-terminus and removal of the OTBS-protecting group from the hydroxytryptophan provided the linear peptide precursor, which could be cyclized to **49** using PyBOP [58] under high dilution conditions and providing good yields. Finally, the benzoyl group had to be removed from the hydroxyleucine and cyclomarin C was purified via preparative HPLC.

**Scheme 11:**
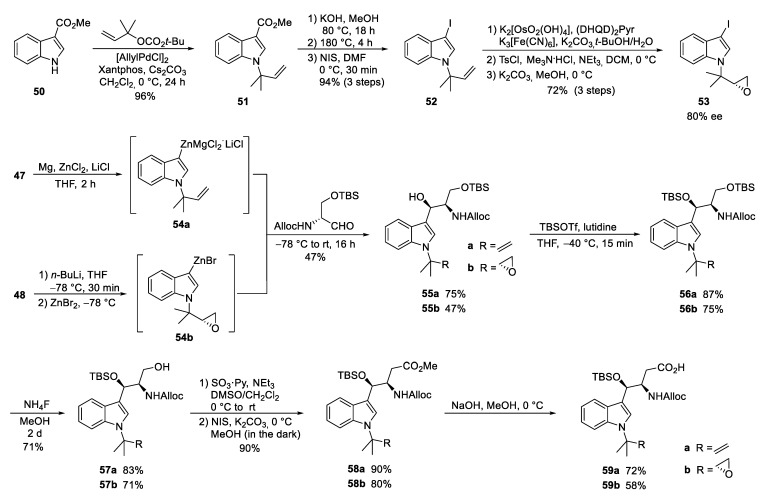
Synthesis of tryptophan derivative **59** (building block ①).

The second synthesis of cyclomarin C and the first for cyclomarin A were reported in 2016 by Barbie and Kazmaier [59]. Both natural products differ only in the oxidation state of the prenylated β-hydroxytryptophan unit ①, which is epoxidized in cyclomarin A. Therefore, a synthetic protocol was developed which gave access to both tryptophan derivatives (Scheme 11). The synthesis started with a relatively new method for regioselective *tert*-prenylation of electron-demanding indoles [60]. Using indole ester **50**, a palladium-catalyzed protocol delivered the required product **51** in almost quantitative yield. At 0 °C, no competitive *n*-prenylation was observed. In the next step, the activating ester functionality needed to be replaced by iodine. Saponification of the ester and heating the neat acid to 180 °C resulted in a clean decarboxylation to the *N*-prenylated indole, which could be iodinated in almost quantitative yield. Iodide **52** was used as a key building block for the synthesis of cyclomarin C, and after epoxidation, cyclomarin A. According to Yokohama et al. [61], **52** was subjected to a Sharpless dihydroxylation, which unfortunately demonstrated only moderate stereoselectivity. The best results were obtained with (DHQD)_2_Pyr as chiral ligand, but the ee did not exceed 80 % [62]. Subsequent tosylation of the primary OH-group and treatment with a base provided a good yield of the desired epoxide **53**. The iodides **52** and **53** were next converted into organometallic reagents and reacted with a protected serinal. While the corresponding Grignard reagents provided only moderate yields and selectivities, zinc reagents were found to be superior. According to Knochel et al. [63,64], **52** was presumably converted into the indole–zinc–magnesium complex **54a**, which was reacted with freshly prepared protected serinal to give the desired *syn*-configured **55a** as a single diastereomer. In the case of the epoxyindole **53**, a slightly different protocol was used. To avoid side reactions during the metalation step, **53** was lithiated at −78 °C with *n*-BuLi and transmetallated with ZnBr_2_ [63,64]. The zinc reagent **54b** was directly reacted with the aldehyde to create **55b**. According to NMR and HPLC, only two diastereomers (ratio 9:1) could be detected following the Sharpless dihydroxylation step. Obviously, the carbonyl addition also here was highly stereoselective. Next, the secondary OH-functionality was TBS-protected under the assumption that a primary OTBS-group could be removed selectively [65]. Notably, only a combination of TBSOTf and lutidine gave the desired product **56a** in high yield, while all other methods failed and resulted in the decomposition of **55a**. Interestingly, no complete conversion was obtained for **55b**, but the silyl ether **56b** was obtained as a single stereoisomer. Obviously, the undesirable diastereomer did not undergo silylation. Next, the primary silyl protecting group was removed using NH_4_F in MeOH [66]. The free alcohols **57** had to be oxidized to the desired carboxylic acids **59**, which were found to be highly sensitive and not very stable. By far, the best results were obtained using a two-step protocol beginning with a Parikh–Doering oxidation [67]. The also very labile aldehydes were directly oxidized to the corresponding methyl esters **58** with *N*-iodosuccinimide in MeOH [68]. These are stable, can be stored under standard refrigeration, and should be saponified to the free acids **59** on demand.

**Scheme 12:**
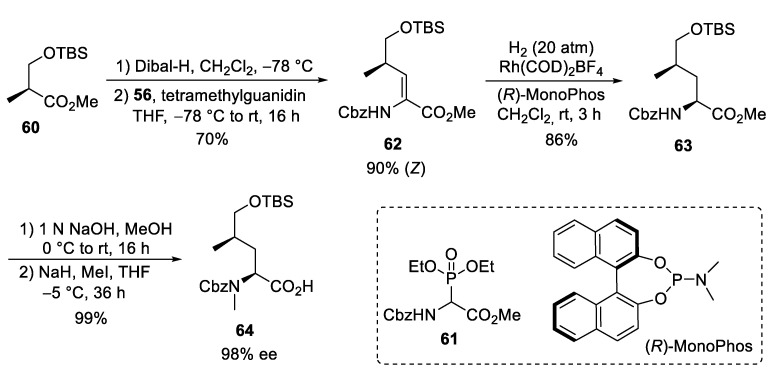
Synthesis of δ-hydroxyleucine derivative 59 (building block ②).

A straightforward protocol was developed for the protected δ-hydroxyleucine ②, starting with the commercially available (*S*)-Roche ester, which was *O*-silylated to **60** (Scheme 12). Subsequent Dibal-H reduction provided the corresponding aldehyde, which was subjected to a Horner–Wadsworth–Emmons reaction using Schmidt’s phosphonoglycinate **61** [69]. The unsaturated amino acid **62** obtained was subjected to asymmetric hydrogenation [70] using (*R*)-MonoPhos as a chiral ligand [71,72]. Subsequent saponification of **63** and *N*-methylation yielded the desired building block **64**.

**Scheme 13:**
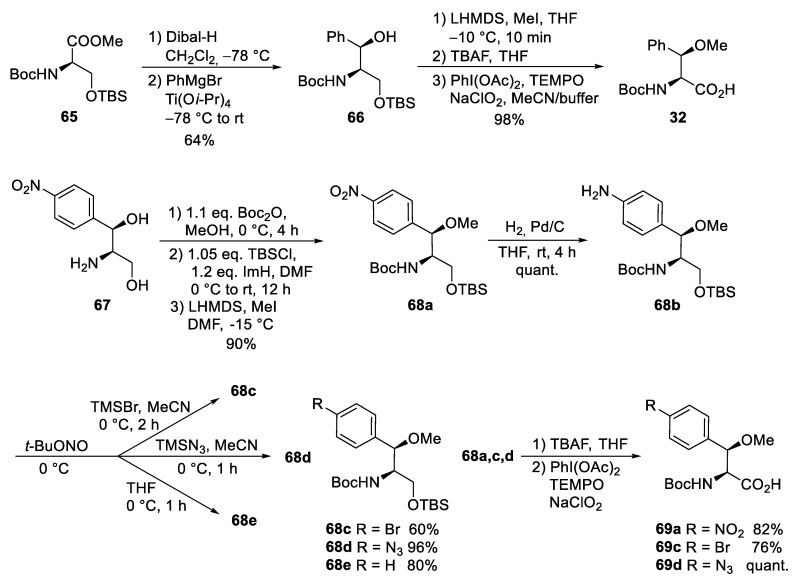
Synthesis of β-methoxyphenylalanine derivatives (building block ④).

The third unusual amino acid, β-methoxyphenylalanine ④, could be obtained similarly to the tryptophan building block ① (Scheme 13). Protected (*R*)-serine **65** was reduced to the corresponding aldehyde, which was subjected to a chelate-controlled aryl-metal addition. The addition of phenylmagnesium bromide provided an acceptable yield but only moderate diastereoselectivity (7:3). In contrast, in the presence of titanium salts, the desired coupling product **66** could be obtained as a single diastereomer in enantiomerically pure form. Further standard transformations yielded building block **32**.

An alternative protocol, which also permitted the synthesis of substituted building blocks, was developed starting from commercially available chloramphenicol base **67** (Scheme 13) [73]. Thus, the amino diol was first converted stepwise into the protected derivative **68a**. The nitro functionality could easily be reduced to the corresponding aniline derivative **68b**, an ideal candidate for further structural variations via diazonium chemistry. Depending on the reaction conditions and additives used, several new derivatives **68c-e** could be obtained, which could be oxidized to the corresponding amino acids **69a-d**, while deamination of **68b** provided the unsubstituted building block **32**.

**Scheme 14:**
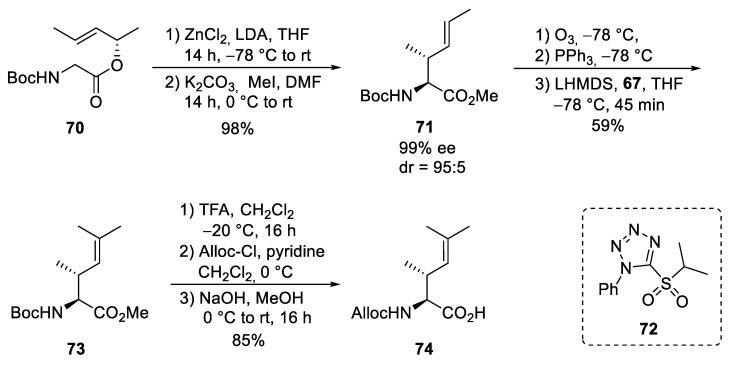
Synthesis of unsaturated amino acid **69** (building block ⑦).

For the synthesis of the fourth, the unsaturated amino acid ⑦, also a chelate enolate Claisen rearrangement, was used starting with chiral ester **70** (Scheme 14) [74,75,76]. Rearrangement of the corresponding zinc enolate proceeded with complete chirality transfer and high stereoselectivity, and ester **71** was obtained after *O*-methylation. Ozonolysis provided the desired aldehyde, which was subjected to a Wittig reaction. However, only tiny amounts of the desired product could be obtained, even with a large excess of the Wittig reagents, which also caused the chiral aldehyde’s epimerization. Better results were obtained with a modified Julia–Kocienski reagent **72**, normally used for (*E*)-selective olefination [77]. Under these conditions, the desired unsaturated building block **73** could be obtained almost epimerization-free. Saponification and change in the *N*-protecting group provided amino acid **74**.

**Scheme 15:**
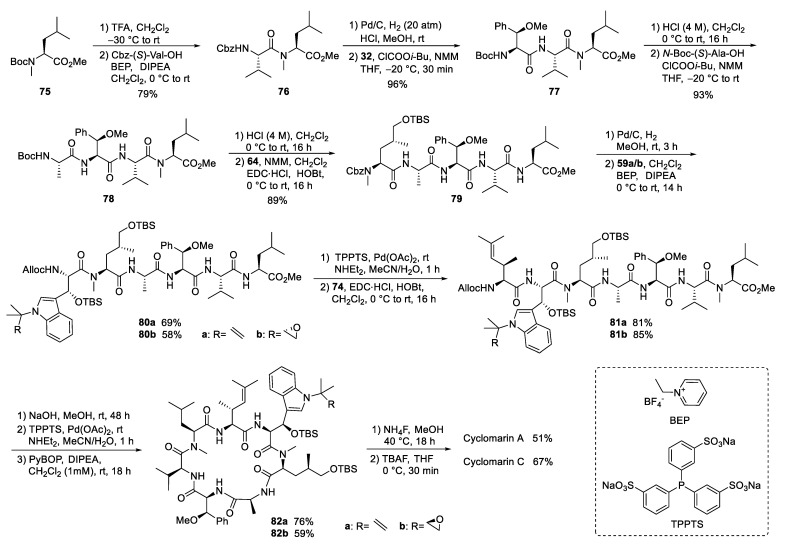
Syntheses of cyclomarin A and C.

With all required building blocks synthesized, the linear synthesis of cyclomarins A and C started with protected *N*-methylleucine **75** (⑥) to cyclize the linear heptapeptide at the same position as achieved by Yao et al. (Scheme 15) [47,56]. The linear strategy was chosen to avoid epimerizations during fragment couplings, and three of the unusual building blocks were incorporated at the end of the synthesis, also allowing for modification of these building blocks and to obtain derivatives for structure-activity relationship (SAR) studies.

The hydrogenolysis of the Cbz-protecting group in dipeptide **76** was not a trivial task. The reaction required an H_2_ pressure of 20 bar to proceed, and two equivalents of HCl had to be added to avoid diketopiperazine formation. The hydrochloride salt was directly coupled with the activated amino acid **32** in the presence of excess base. The next steps were standard peptide couplings. BEP [78,79] was used to incorporate the sensitive tryptophan building blocks **59**. The Alloc protecting group was removed, Pd-catalyzed, and the linear heptapeptide was cyclized using Yao’s protocol [47,56]. Finally, a two-step protocol was needed to remove the two OTBS protecting groups separately, providing good yields of cyclomarins A and C. Notably, cyclomarin D (desmethylcyclomarin C), missing only the *N*-methyl group of the δ-hydroxyleucin, was also obtained by this protocol [80].

## 5. Syntheses of Cyclomarin Derivatives

Given the outstanding biological properties of the cyclomarins, it is not surprising that multiple investigations have been undertaken to obtain modified cyclomarins for SAR studies.

### 5.1. Mutasyntheses of Cyclomarin Derivatives

**Scheme 16:**
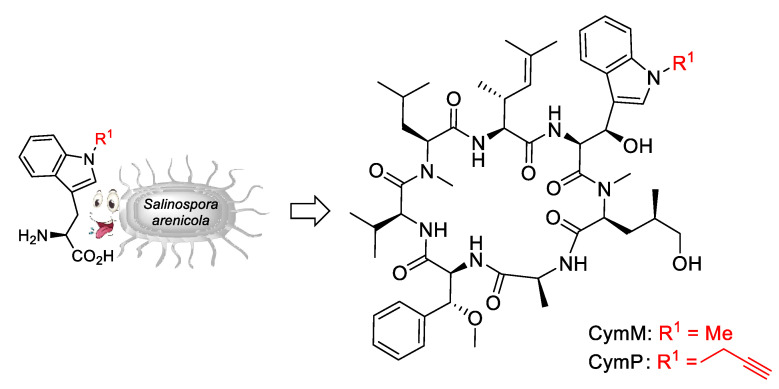
Synthesis of cyclomarin derivatives via mutasyntheses.

In studies of cyclomarin biosynthesis, Moore et al. identified an *N*-prenyltransferase to prenylate tryptophan prior to loading the modified amino acid onto the NRPS [35]. A knockout mutant of *Salinospora arenicola* CNS-205 with a disrupted prenyltransferase gene failed to produce cyclomarins but did produce desprenylcyclomarin C, although at a 100-fold lower production rate. Obviously, tryptophan is not a good substrate for the cyclomarin NRPS, but other *N*-alkylated tryptophans are well accepted [34]. These can be obtained by simple alkylation of suitably protected tryptophan derivatives [81]. Feeding the bacteria with methylated and propargylated tryptophan resulted in the production of new cyclomarin derivatives CymM and CymP (Scheme 16).

### 5.2. Semisyntheses of Cyclomarin Derivatives

**Scheme 17:**
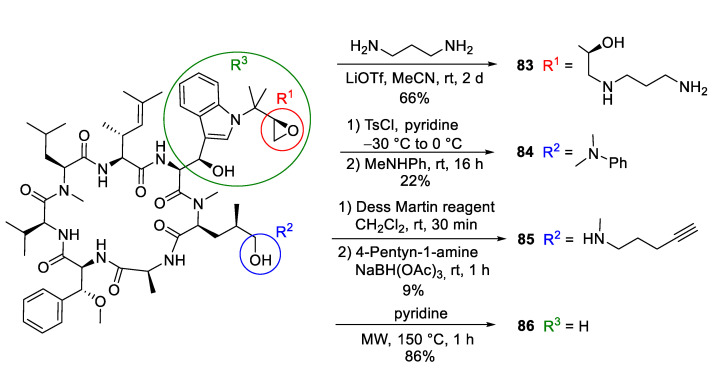
Synthesis of cyclomarin derivatives via semisyntheses.

Researchers at Novartis observed that cyclomarin A might be a good candidate for drug development since it kills not only *Mycobacterium tuberculosis* (Mtb) but also the malaria parasite *Plasmodium falciparum* [82]. Thus, cyclomarin A is a rare example of a natural product with two distinct and specific modes of action. The researchers modified cyclomarin A, obtained by fermentation (Scheme 17) [83]. Ring-opening of the prenylepoxide with 1,3-propanediamine provided amine **83**, which was linked to sepharose beads and processed by affinity chromatography to identify the target. Interestingly, **83** inhibits the growth of Mtb similarly to cyclomarin A [83,84]. Obviously, the epoxide of the *N*-prenyl side chain is not responsible for the biological activity, at least against Mtb. In contrast, replacing the terminal OH-group of the δ-hydroxyleucine ② by an *N*-methylanilin (**84**) caused a significant drop (>200 fold) in activity [83].

Notably, the amine **77**, which was very active against Mtb, demonstrated little activity against *Plasmodium falciparum*. Therefore, the Novartis researchers also synthesized acetylenic amine **85** for target fishing [82]. This compound was almost as active as cyclomarin A in biochemical assays toward the biological target PfAp3Aase. As an inactive control, a “minimized” cyclomarin **86** with a missing tryptophan side chain was used. This compound was obtained by retro aldol reaction.

### 5.3. Total Synthesis of Cyclomarin Derivatives

**Figure 4:**
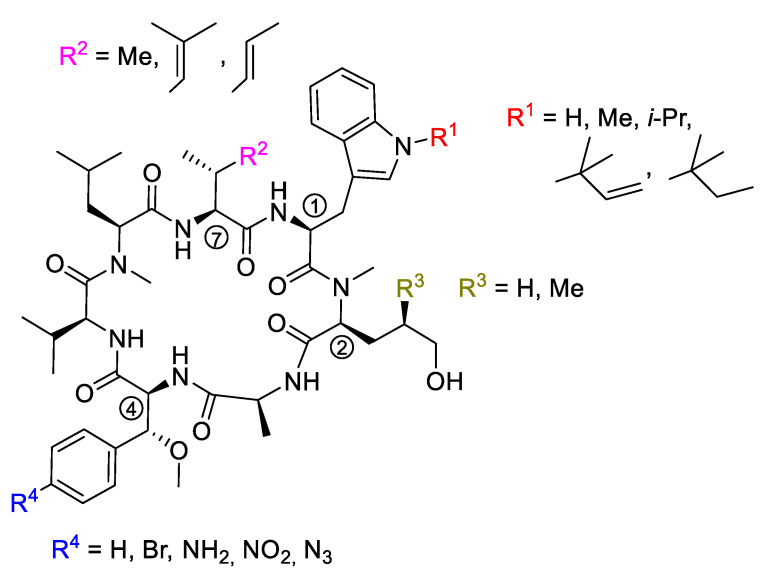
Desoxycyclomarins obtained by total syntheses.

Retro aldol reaction of the β-hydroxytryptophan seems to be a serious stability issue, also during synthesis. This building block undergoes the discussed side reaction proceeding under slightly basic conditions. Under acidic conditions, water is rapidly eliminated, resulting in the formation of the α,β-unsaturated dehydrotryptophan derivative. To avoid these problems, Kazmaier et al. synthesized a series of cyclomarin derivatives containing non-hydroxylated tryptophans (desoxycyclomarins), e.g., the building blocks found in ilamycins/rufomycin [85,86]. *N*-Isopropyltryptophan was obtained via Negishi coupling of 3-iodo-*N*-isopropylindole with protected zincated iodoalanine [86]. Other derivatives can be obtained by an improved protocol for tryptophan alkylations [81]. Several modifications have also been made on the β-methoxyphenylalanine unit ④ [73]. Other derivatives were synthesized utilizing further modifications on building blocks ② and ⑦ (Figure 4).

## 6. Biological Activities and Mode of Action

### 6.1. Biological Activities of Ilamycins/Rufomycins

Both the ilamycins [14,15] and rufomycins [16,17] were isolated independently from *Streptomycetes* in 1962 as new antibiotics, active against acid-fast bacteria, especially *Mycobacteria*. Ilamycin A was reported to inhibit *Mycobacterium* 607 at 0.5 μg/mL, while ilamycin B was less active (3 μg/mL). The rufomycins were reported to be highly active against *Mycobacterium smegmatis* (RufA: 0.2 μg/mL, RufB: 0.5 μg/mL) and *Mycobacterium tuberculosis* (RufA: 0.1–0.4 μg/mL, RufB: 1–5 μg/mL), also strains resistant to other antibiotics such as streptomycin (SM), neomycin (NM), kanamycin (KM), and isonicotinic acid hydrazide (INHA. The compounds are almost inactive against other Gram-positive and Gram-negative bacteria, fungi, and yeasts. In addition, no significant toxicity was observed on four-week-old mice by intraperitoneal injection (Ruf A, LD_0_ 200 mg/kg and LD_100_ 360 mg/kg) [16].

**Figure 5:**
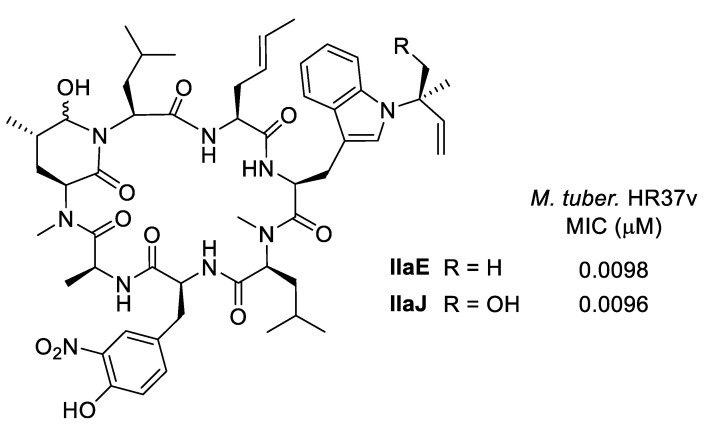
Most active ilamycins.

Ma and Ju et al. recently isolated 12 new ilamycin analogs (IlaG-R) from a 200 L scale culture of mutant *Streptomyces atratus* ZH16 ΔilaR. The analogs demonstrated a slightly different oxidation pattern compared to the previously isolated ilamycins [27,28]. Most derivatives showed the same antibacterial activity as the other ilamycins and rufomycins with MIC’s in the range of 1−2 μM against *Mycobacterium tuberculosis*, while the most active examples thus far have been ilamycin E and J (Figure 5), both more active than rifampicin used as a positive control.

Based on the bioassay data, some structure-activity relationships became evident. Cyclized compounds such as IlaE and IlaJ demonstrated greater activity than open-chain leucine derivatives such as IlaB, IlaD, or IlaF (Figure 1). Oxidation of the prenyl side chain did not affect activity. The nitro group on the tyrosine seems to play an important role [27,28].

**Figure 6:**
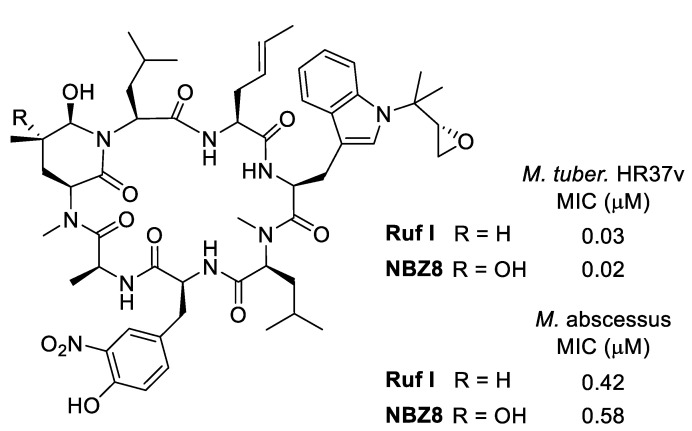
Most active rufomycins.

In 2020, Pauli et al. isolated eight new rufomycins (rufNBZ1-NBZ8) together with five already known derivatives from the *Streptomyces atratus* strain MJM3502 [29]. Analogous to the ilamycins, the most active derivatives contain a cyclic oxidized leucine such as RufNBZ8 or the previously isolated Ruf I (Figure 6) with an MIC (*M. tuber.* HR37v) similar to rifampicin (0.03 μM). Ruf I maintained activity against monoresistant, MDR, and XDR strains, indicating that the Rufs most likely have a different molecular target than current drugs used in TB therapy. Additionally, Ruf I retained its activity against strains from the five global *M. tuberculosis* clades representative of clinical TB disease worldwide [87]. Especially interesting is its activity against *M. abscessus*, one of the more difficult-to-treat mycobacteria [88].

Detailed studies indicated that the ilamycins/rufomycins inhibit ClpC1, an enzyme currently not targeted for TB treatment. ClpC1 is the ATP-dependent homolog of the ClpC class of chaperone proteins present in *M. tuberculosis* [89] and is highly conserved among mycobacteria. Unlike in many other bacteria, ClpC proteins are essential for the viability of mycobacteria, especially *M. tuberculosis*. In this species, the strictly regulated ClpC1 associates with the proteolytic domains, ClpP1 and ClpP2, and together they are responsible for waste protein degradation within the cell [90]. Without functional ClpC1, cellular protein degradation is reduced or stopped completely. Ruf I significantly decreases the proteolytic capabilities of the ClpC1/P1/P2 complex while having no significant effect on the ATPase activity of ClpC1 [87]. In this respect, it differs from ecumicin (Ecu), another anti-tubercular cyclic peptide, which inhibits ClpC1 but stimulates ATPase activity. Details of the binding topology and chemical mode of (inter)action of these cyclopeptides were reported by Cho et al. [91]. The X-ray structure of a complex of the *N*-terminal helical domain (NTD) of ClpC1 and Ruf I reveals distinct differences from the corresponding ClpC1-NTD-cyclomarin A structure. Surprisingly, the complex structure shows that the epoxide moiety of Ruf I is opened and covalently bound to ClpC1-NTD via the sulfur atom of a methionine in the binding pocket [92,93].

Although the anti-tubercular activity is the most prominent effect of ilamycins/rufomycins, certain derivatives show some anti-cancer activities, while others do not. IlaC (Ruf I) was found to be preferentially cytotoxic towards triple-negative breast cancer (TNBC) cell lines, the most aggressive subtype of breast cancer with poor prognoses. IlaC can induce Bax/Bcl-2-related caspase-dependent apoptosis and inhibit migration and invasion in TNBC by suppressing IL-6-induced STAT3 phosphorylation [94]. IlaE, but not IlaF, decreases cell viability, inhibits G1/S cell cycle progression, and promotes apoptosis in the TNBC cell lines HCC1937 (IC50 14.2 μM) and MDA-MB-468 (IC50 24.5 μM). It promotes apoptosis via activation of endoplasmic reticulum (ER) stress, increasing CHOP expression and down-regulating the expression of anti-apoptotic protein Bcl-2 [94].

### 6.2. Biological Activities of Cyclomarins

Fenical and Clardy et al., who isolated the cyclomarins A−C in 1999, reported that the major metabolite cyclomarin A (CymA) showed anti-inflammatory activity in vivo and in vitro [30]. Lateron, Pittayakhajonwut et al. reported that CymC shows anti-malarial activity against multidrug-resistant *Plasmodium falciparum* strains (IC_50_ 0.25 μM) as well as anti-TB activity (MIC: 0.1 μM) [95]. No significant activity was observed against cancer cells or *Candida albicans* (IC_50_ > 50 μM). Schmitt et al. at Novartis investigated the mode of action in detail [83]. CymA was found to bind specifically and with a high affinity to ClpC1 and did not interfere with ATP binding by the two ATPase domains of ClpC1. The antimycobacterial activity of cyclomarin derivatives correlates well with binding to ClpC1. For example, amino alcohol **83** (Scheme 17) demonstrated high affinity (MIC: 0.1 μM), while no binding was observed for the inactive amine **84**. The hydroxy functionality on leucine ② is clearly important for binding, but not the epoxide.

The exact binding of CymA toward the NTD of ClpC1 was determined by high-resolution co-crystal structure analysis [84]. The overall sequence identity of ClpC1 from various *Mycobacterium* species is close to 95%, but the NTD of mycobacterial ClpC1 is 100% conserved. This phenomenon explains why all tested mycobacteria were found to be sensitive to CymA. Based on the structure of the complex, several mutations were engineered into ClpC1, which showed reduced CymA binding in vitro. The ClpC1 mutants were overexpressed in mycobacteria and two showed resistance to CymA, providing clear evidence that ClpC1 is the target of CymA.

Using NMR and small-angle X-ray scattering, Schanda and Fraga et al. demonstrated that arginine-phosphate binding to the ClpC1 NTD induces millisecond dynamics [96]. Cyclomarin binding to this domain specifically blocks these dynamics. Based on these results, a proposed mechanism of action involves the cyclomarin-induced restriction of ClpC1 dynamics, which modulates the chaperone enzymatic activity leading eventually to cell death [96]. Very recently, Mogk et al. showed that CymA activates an ATP-driven bacterial AAA+ protease (e.g., ClpP) and that cell death is induced by uncontrolled proteolytic activity of these enzymes [97].

However, anti-TB activity is not the only interesting feature of the cyclomarins. Schmitt et al. showed that CymA is a potent growth inhibitor of *Plasmodium falciparum*, and its molecular target, diadenosine triphosphate hydrolase (PfAp3Aase), was identified by chemical proteomics [82]. CymA is a specific inhibitor of the plasmodial enzyme (IC_50_: 0.004 μM) but not of the closest human homolog hFHIT (IC_50_ > 10 μM). Co-crystallization experiments demonstrated a unique inhibitor binding mode. One molecule of CymA binds a dimeric PfAp3Aase and prevents the formation of the enzyme-substrate complex. These results validate PfAp3Aase as a new drug target for the treatment of malaria. Thus, CymA is a rare example of a natural product with two distinct and specific modes of action.

Unfortunately, CymA as a natural product lacks satisfactory pharmacokinetic properties, making it challenging for optimization into an (orally) bioavailable drug. Therefore, Kazmaier et al. tried to simplify the complex structure of the cyclomarins without losing significant biological activity. Because the β-hydroxytryptophan unit ① is the most critical building block, they removed the hydroxy functionality completely, and the desoxycyclomarins obtained were further modified on the prenyl side chain of ①, the hydroxylated leucine ②, the β-methoxyphenylalanine ④ and the unsaturated amino acid ⑦ [37,73,85,86]. Selected biological data are summarized in Table 1.

Certain modifications at R^1^ were well-tolerated (series **87**). Reduction to an isopropyl group (**87c**) provides an especially promising simplification retaining antimycobacterial activity. In general, manipulations at this position do not result in dramatic effects on potency measured against *Mtb* and *Pfalcp*. Interestingly, the methyl group in **87d** is an appropriate balance between reducing synthetic complexity and loss of activity.

Results obtained for the modifications at R^2^ were consistent with the data obtained by X-ray structure analysis [82]. In the case of *Pfalcp*, where this residue is completely buried between the target and the ligand scaffold, large changes are not accepted. However, it is important that removing the terminal methyl group in the *cis* position of the γ,δ-unsaturated side chain led to an equivalent or even slightly improved activity. Further simplifications, however, are not advisable, as they lead to dramatic activity losses, which are seen in the comparison of **87a** and **88a**. In contrast, the anti-TB activity was not influenced.

Removal of the methyl group at R^3^ led to a decrease in potency against both pathogens (**89**). This result was indeed expected for the Mtb target ClpC1-NTD [96]. This group interacts well with the target, which is also closely packed with the indole motif of the tryptophan core. However, it is not yet clear why deletion of this methyl residue impairs activity towards *Pfalcp*.

Modifications of R^4^ on β-methoxyphenylalanine ④ were also well tolerated (**90**). The amino- and azido-derivatives **90a** and **90b** were equipotent to cyclomarin C, and the nitro compound **90c** was even twice as active. Only in the case of bromo derivative **90d** was a significant drop in activity observed.

## 7. Conclusions

The ilamycins/rufomycins and the cyclomarins are highly interesting marine cycloheptapeptides characterized by their incorporation of unusual amino acids. The natural products are produced by *Streptomyces* sp. and show potent activity against a range of mycobacteria, including multidrug-resistant strains of *Mycobacterium tuberculosis*. No significant activity has been observed towards other Gram-positive and Gram-negative bacteria or fungi.

The cyclomarins are also very potent inhibitors of *Plasmodium falciparum*, the organism that causes malaria. Biosynthetically, the cyclopeptides are obtained via a heptamodular NRPS that directly incorporates some of the nonproteinogenic amino acids, while oxidations at certain positions allow the compounds to proceed to protein-bound biosynthetic intermediates. Cyclized ilamycins/rufomycins are obtained by oxidative post-NRPS cyclization of leucine ⑦, the last introduced amino acid in the biosynthesis. A wide range of derivatives can be obtained by fermentation, while bioengineering also allows the mutasynthesis of derivatives, especially cyclomarins. Other derivatives are accessible by semisynthesis or total syntheses, reported for both natural product classes. Some of these derivatives were used to identify the biological targets of these peptides. The anti-TB activity results from the binding of the peptides to the *N*-terminal domain (NTD) of the protease ClpC1, causing cell death by the uncontrolled proteolytic activity of associated enzymes.

Diadenosine triphosphate hydrolase (PfAp3Aase) was found to be the active target of the cyclomarins in Plasmodia, and this enzyme might be a good candidate for the treatment of malaria. SAR studies of natural and synthetic derivatives on the ilamycins/rufomycins and cyclomarins indicate which parts of the molecules can be simplified/modified without losing activity towards either target.

## Figures and Tables

**Table 1:** Biological data of cyclomarins and selected desoxycyclomarins.

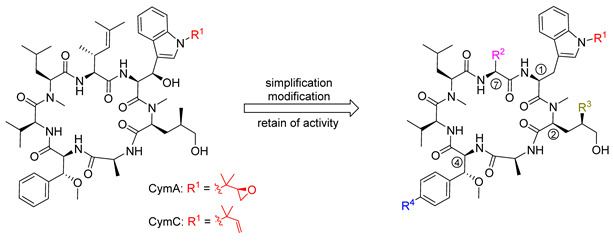							
Compound	R^1^	R^2^	R^3^	R^4^	Mtb	Pfalcp	
					wt Erdman MIC[µM]	3D7 IC_50_[nM]	Dd2 IC_50_[nM]
Isoniazid					0.9		
CQ						3.4	233.9
Cyclomarin A	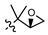	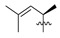	Me	H	0.125	36.8	27.7
Cyclomarin C	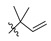	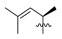	Me	H	0.25	42.8	
Desoxycyclomarin C	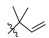	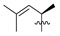	Me	H	0.9	39.8	
**87a**	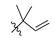	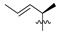	Me	H	0.5	9.0	12.9
**87b**	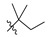	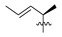	Me	H	1.7	4.4	6.5
**87c**	*i*Pr	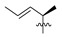	Me	H	0.4	47.8	76.0
**87d**	Me	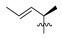	Me	H	0.9	13.4	17.8
**87e**	H	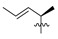	Me	H	1.5	28.1	27.5
**88a**	*i*Pr	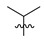	Me	H	0.4	355.7	300.3
**88b**	Me	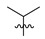	Me	H	1.6	230.3	256.7
**88c**	H	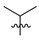	Me	H	0.5	314.2	421.5
**89a**	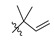	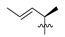	H	H	2.3	47.9	36.7
**89b**	*i*Pr	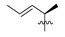	H	H	1.8	177.5	287.8
**89c**	Me	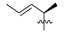	H	H	3.4	362.4	318.4
**90a**	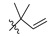	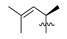	Me	NH_2_	0.25		
**90b**	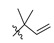	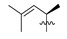	Me	N_3_	0.26		
**90c**	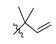	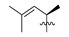	Me	NO_2_	0.13		
**90d**	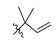	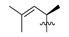	Me	Br	4.1		

## Data Availability

Not applicable.

## References

[1] Carroll A.R., Copp B.R., Davis R.A., Keyzers R.A., Prinsep M.R. (2020). Marine natural products. Nat. Prod. Rep..

[2] Carroll A.R., Copp B.R., Davis R.A., Keyzers R.A., Prinsep M.R. (2021). Marine natural products. Nat. Prod. Rep..

[3] Barreca M., Spane V., Montalbano A., Cueto M., Marrero A.R.D., Deniz I., Erdogan A., Bilela L.L., Moulin C., Taffin-de-Givenchy E. (2020). Marine Anticancer Agents: An Overview with a Particular Focus on Their Chemical Classes. Mar. Drugs.

[4] McCauley E.P., Pina I.C., Thompson A.D., Bashir K., Weinberg M., Kurz S.L., Crews P. (2020). Highlights of marine natural products having parallel scaffolds found from marine-derived bacteria, sponges, and tunicates. J. Antibiot..

[5] Qamar H., Hussain K., Soni A., Khan A., Hussain T., Chenais B. (2021). Cyanobacteria as natural therapeutics and pharmaceutical potential: Role in antitumor activity and as nanovectors. Molecules.

[6] Njoroge M., Njuguna N.M., Mutai P., Ongarora D.S.B., Smith P.W., Chibale K. (2014). Recent approaches to chemical discovery and development against malaria and the neglected tropical diseases human african trypanosomiasis and schistosomiasis. Chem. Rev..

[7] WHO. https://www.who.int/health-topics/tuberculosis#tab=tab_1.

[8] De Opitz C.L.M., Sass P. (2020). Tackling antimicrobial resistance by exploring new mechanisms of antibiotic action. Future Microbiol..

[9] Dooley K.E., Park J.G., Swindells S., Allen R., Haas D.W., Cramer Y., Aweeka F., Wiggins I., Gupta A., Lizak P. (2012). Safety, tolerability, and pharmacokinetic interactions of the antituberculous agent TMC207 (bedaquiline) with efavirenz in healthy volunteers: AIDS clinical trials group study A5267. J. Acq. Imm. Def..

[10] Zumla A., Nahid P., Cole S.T. (2013). Advances in the development of new tuberculosis drugs and treatment regimens. Nat. Rev. Drug Discov..

[11] Igarashi M., Ishizaki Y., Takahashi Y. (2018). New antituberculous drugs derived from natural products: Current perspectives and issues in antituberculous drug development. J. Antibiot..

[12] Lee H., Suh J.W. (2016). Anti-tuberculosis lead molecules from natural products targeting *Mycobacterium tuberculosis* ClpC1. J. Ind. Microbiol. Biotechnol..

[13] Newman D.J., Cragg G.M. (2012). Natural products as sources of new drugs over the 30 years from 1981 to 2010. J. Nat. Prod..

[14] Nakayama Y., Takita T., Ozawa H., Umezawa H., Tahara K. (1962). Studies on ilamycin. J. Antibiot..

[15] Takita T., Ohi K., Maeda K., Okami Y., Umezawa H. (1962). New antibiotics, ilamycins. J. Antibiot..

[16] Higashidani E., Ueyanagi J., Shibata M., Nakazawa K., Miyake A., Iwasaki H., Yamamoto H. (1962). Studies on *Streptomycetes*. 2. Rufomycin A and A, new antituberculous antibiotics. Agric. Biol. Chem..

[17] Shibata M., Yamamoto H., Higashidani E., Nakazawa K. (1962). Studies on *Streptomycetes*. 1. *Streptromyces atratus* nov. sp. producing new antituberculous antibiotics rufomycin A and B. Agric. Biol. Chem..

[18] Cary L.W., Takita T., Ohnishi M. (1971). Study of secondary structure of ilamycin-B1 by 300 MHz proton magnetic resonance. FEBS Lett..

[19] Iitaka Y., Nakamura H., Takada K., Takita T. (1974). X-RAY study of ilamycin B1, a cyclic heptapeptide antibiotic. Acta Cryst. B.

[20] Iwasaki H., Witkop B. (1964). New methods for nonenzymatic peptide cleavage. Electrolytic differential + solvolytic cleavage of antibiotic cyclopeptide rufymacin. J. Am. Chem. Soc..

[21] Takita T. (1963). Amino acid sequence of ilamycin and ilamycin B. J. Antibiot..

[22] Takita T., Maeda K., Naganawa H., Umezawa H. (1964). Structures of ilamycin and ilamycin B2. J. Antibiot..

[23] Takita T., Naganawa H., Maeda K., Umezawa H. (1965). The structural diffference among ilamycin, ilamycin C1 and ilamycin C2. J. Antibiot..

[24] Fujino M., Kamiya T., Miyake A., Iwasaki H., Ueyanagi J. (1964). Tryptophan moiety of rufomycin homologs. Chem. Pharm. Bull..

[25] Takita T., Naganawa H., Maeda K., Umezawa H. (1964). Further studies on tryptophan parts of ilamycins. J. Antibiot..

[26] Takita T., Naganawa H. (1963). L-2-Amino-4-hexenoic acid in ilamycins. J. Antibiot..

[27] Ma J.Y., Huang H.B., Xie Y.C., Liu Z.Y., Zhao J., Zhang C.Y., Jia Y.X., Zhang Y., Zhang H., Zhang T.Y. (2017). Biosynthesis of ilamycins featuring unusual building blocks and engineered production of enhanced anti-tuberculosis agents. Nat. Commun..

[28] Sun C.L., Liu Z.Y., Zhu X.C., Fan Z.Y., Huang X.M., Wu Q.L., Zheng X.H., Qin X.J., Zhang T.Y., Zhang H. (2020). Antitubercular ilamycins from marine-derived *Streptomyces atratus* SCSIO ZH16 Δ ilaR. J. Nat. Prod..

[29] Zhou B., Shetye G., Yu Y., Santarsiero B.D., Klein L.L., Abad-Zapatero C., Wolf N.M., Cheng J., Jin Y., Lee H. (2020). Antimycobacterial rufomycin analogues from *Streptomyces atratus* strain MJM3502. J. Nat. Prod..

[30] Renner M.K., Shen Y.C., Cheng X.C., Jensen P.R., Frankmoelle W., Kauffman C.A., Fenical W., Lobkovsky E., Clardy J. (1999). Cyclomarins A-C, new antiinflammatory cyclic peptides produced by a marine bacterium (*Streptomyces* sp.). J. Am. Chem. Soc..

[31] Kumamoto T., Koshino H., Watanabe D., Matsumoto Y., Aoyama K., Harada K., Ishikawa T., Mikami Y. (2010). M10709, a new peptide antibiotic from clinically isolated *Streptomyces* sp.. Heterocycles.

[32] Li L., MacIntyre L.W., Ali T., Russo R., Koirala B., Hernandez Y., Brady S.F. (2021). Biosynthetic interrogation of soil metagenomes reveals metamarin, an uncommon cyclomarin congener with activity against *Mycobacterium tuberculosis*. J. Nat. Prod..

[33] Tomita H., Katsuyama Y., Minami H., Ohnishi Y. (2017). Identification and characterization of a bacterial cytochrome P450 monooxygenase catalyzing the 3-nitration of tyrosine in rufomycin biosynthesis. J. Biol. Chem..

[34] Schultz A.W., Lewis C.A., Luzung M.R., Baran P.S., Moore B.S. (2010). Functional characterization of the cyclomarin/cyclomarazine prenyltransferase CymD directs the biosynthesis of unnatural cyclic peptides. J. Nat. Prod..

[35] Schultz A.W., Oh D.C., Carney J.R., Williamson R.T., Udwary D.W., Jensen P.R., Gould S.J., Fenical W., Moore B.S. (2008). Biosynthesis and structures of cyclomarins and cyclomarazines, prenylated cyclic peptides of marine actinobacterial origin. J. Am. Chem. Soc..

[36] He J.Q., Wei X., Yang Z.J., Li Y., Ju J.H., Ma J.Y. (2020). Characterization of regulatory and transporter genes in the biosynthesis of anti-tuberculosis ilamycins and production in a heterologous host. Mar. Drugs.

[37] Kiefer A., Kazmaier U. (2019). Syntheses of cyclomarins—Interesting marine natural products with distinct mode of action towards malaria and tuberculosis. Synthesis.

[38] Cheng Y.Y., Tang S.B., Guo Y., Ye T. (2018). Total synthesis of anti-tuberculosis natural products ilamycins E-1 and F. Org. Lett..

[39] Luzung M.R., Lewis C.A., Baran P.S. (2009). Direct, chemoselective *N-tert*-prenylation of indoles by C-H functionalization. Angew. Chem. Int. Ed..

[40] Arda A., Soengas R.G., Nieto M.I., Jimenez C., Rodriguez J. (2008). Total synthesis of (-)-dysithiazolamide. Org. Lett..

[41] Hanessian S., Margarita R. (1998). 1,3-asymmetric induction in dianionic allylation reactions of amino acid derivatives-synthesis of functionally useful enantiopure glutamates, pipecolates and pyroglutamates. Tetrahedron Lett..

[42] Padron J.M., Kokotos G., Martin T., Markidis T., Gibbons W.A., Martin V.S. (1998). Enantiospecific synthesis of alpha-amino acid semialdehydes: A key step for the synthesis of unnatural unsaturated and saturated alpha-amino acids. Tetrahedron Asymmetry.

[43] Schöllkopf U., Groth U., Deng C. (1981). Asymmetric synthesis via heterocyclic intermediates. 6. Enantioselective synthesis of (*R*)-amino acids using valine as a chiral agent. Angew. Chem. Int. Ed. Engl..

[44] Albericio F., Cases M., Alsina J., Triolo S.A., Carpino L.A., Kates S.A. (1997). On the use of PyAOP, a phosphonium salt derived from HOAt, in solid-phase peptide synthesis. Tetrahedron Lett..

[45] Chen S.Q., Xu J.C. (1991). Pentafluorophenyl diphenylphosphinate—A new efficient coupling reagent in peptide chemistry. Tetrahedron Lett..

[46] Sathish K., Reddy G.P.K., Mainkar P.S., Chandrasekhar S. (2011). Synthesis of the ‘southern’ tripeptide of Cyclomarins A and C having novel anti-tuberculocidal mode of action. Tetrahedron Asymmetry.

[47] Wen S.J., Yao Z.J. (2004). Total synthesis of cyclomarin C. Org. Lett..

[48] Wen S.J., Zhang H.W., Yao Z.J. (2002). Synthesis of a fully protected (2*S*,3*R*)-*N*-(1′,1′-dimethyl-2′-propenyl)-3-hydroxytryptophan from tryptophan. Tetrahedron Lett..

[49] Tao B., Schlingloff G., Sharpless K.B. (1998). Reversal of regioselection in the asymmetric aminohydroxylation of cinnamates. Tetrahedron Lett..

[50] Barbie P., Kazmaier U. (2015). Synthesis of fully protected, reverse *N*-prenylated (2*S*,3*R*)-3-hydroxytryptophan, a unique building block of the cyclomarins. Org. Biomol. Chem..

[51] Evans D.A., Ripin D.H.B., Halstead D.P., Campos K.R. (1999). Synthesis and absolute stereochemical assignment of (+)-miyakolide. J. Am. Chem. Soc..

[52] Easton C.J., Hutton C.A., Roselt P.D., Tiekink E.R.T. (1991). Synthesis and molecular structure of stable derivatives of (*E*)-dehydrophenylalanine and (*Z*)-dehydrophenylalanine. Aust. J. Chem..

[53] Easton C.J., Hutton C.A., Roselt P.D., Tiekink E.R.T. (1994). Stereocontrolled synthesis of β-hydroxyphenylalanine and β-hydroxytyrosine derivatives. Tetrahedron.

[54] Kazmaier U., Krebs A. (1995). Synthesis of chiral γ,δ-unsaturated amino acids by asymmetric ester enolate Claisen rearrangement. Angew. Chem. Int. Ed. Engl..

[55] Kazmaier U., Mues H., Krebs A. (2002). Asymmetric chelated Claisen rearrangements in the presence of chiral ligands—Scope and limitations. Chem. Eur. J..

[56] Wen S.J., Hu T.S., Yao Z.J. (2005). Macrocyclization studies and total synthesis of cyclomarin C, an anti-inflammatory marine cyclopeptide. Tetrahedron.

[57] Cabre J., Palomo A.L. (1984). New experimental strategies in amide synthesis using *N*,*N*-Bis-2-oxo-3-oxazolidinyl phosphordiamidic chloride. Synthesis.

[58] Coste J., Lenguyen D., Castro B. (1990). PYBOP—A new peptide coupling reagent devoid of toxic byproducts. Tetrahedron Lett..

[59] Barbie P., Kazmaier U. (2016). Total Synthesis of Cyclomarin A, a Marine Cycloheptapeptide with Anti-Tuberculosis and Anti-Malaria Activity. Org. Lett..

[60] Johnson K.F., Van Zeeland R., Stanley L.M. (2013). Palladium-catalyzed synthesis of *N*-*tert*-prenylindoles. Org. Lett..

[61] Sugiyama H., Shioiri T., Yokokawa F. (2002). Syntheses of four unusual amino acids, constituents of cyclomarin A. Tetrahedron Lett..

[62] Della Sala G., Izzo I., Spinella A. (2006). A Pd-mediated approach to the synthesis of an unusual β-hydroxytryptophan amino acid constituent of cyclomarin A. Synlett.

[63] Metzger A., Bernhardt S., Manolikakes G., Knochel P. (2010). MgCl_2_-accelerated addition of functionalized organozinc reagents to aldehydes, ketones, and carbon dioxide. Angew. Chem. Int. Ed..

[64] Piller F.M., Metzger A., Schade M.A., Haag B.A., Gavryushin A., Knochel P. (2009). Preparation of polyfunctional arylmagnesium, arylzinc, and benzylic zinc reagents by using magnesium in the presence of LiCl. Chem. Eur. J..

[65] Corey E.J., Venkateswarlu A. (1972). Protection oh hydroxyl groups as *tert*-Butyldimethylsilyl derivatives. J. Am. Chem. Soc..

[66] Futagawa S., Inui T., Shiba T. (1973). Nuclear magnetic-resonance study of stereoisomeric 2-oxazolidone and 2-phenyl-2-oxazoline derivatives of α-amino-β-hydroxy acids. Bull. Chem. Soc. Jpn..

[67] Parikh J.R., Doering W.V.E. (1967). Sulfur trioxide in oxidation of alcohols by dimethyl sulfoxide. J. Am. Chem. Soc..

[68] McDonald C., Holcomb H., Kennedy K., Kirkpatrick E., Leathers T., Vanemon P. (1989). *N*-iodosuccinimide-mediated conversion of aldehydes to methyl esters. J. Org. Chem..

[69] Schmidt U., Griesser H., Leitenberger V., Lieberknecht A., Mangold R., Meyer R., Riedl B. (1992). Amino-acids and peptides. 81. Diastereoselective formation of (*Z*)-didehydroamino acid-esters. Synthesis.

[70] Schmidt U., Lieberknecht A., Kazmaier U., Griesser H., Jung G., Metzger J. (1991). Amino-acids and peptides. 75. Synthesis of dihydroxyamino and trihydroxyamino acids—Construction of lipophilic tripalmitoyldihydroxy-α-amino acids. Synthesis.

[71] Panella L., Aleixandre A.M., Kruidhof G.J., Robertus J., Feringa B.L., de Vries J.G., Minnaard A.J. (2006). Enantioselective Rh-catalyzed hydrogenation of *N*-formyl dehydroamino esters with monodentate phosphoramidite ligands. J. Org. Chem..

[72] Van den Berg M., Minnaard A.J., Schudde E.P., van Esch J., de Vries A.H.M., de Vries J.G., Feringa B.L. (2000). Highly enantioselective rhodium-catalyzed hydrogenation with monodentate ligands. J. Am. Chem. Soc..

[73] Kiefer A., Kazmaier U. (2019). Synthesis of modified β-methoxyphenylalanines via diazonium chemistry and their incorporation in desoxycyclomarin analogues. Org. Biomol. Chem..

[74] Kazmaier U., Schneider C. (1996). Stereoselective synthesis of unsaturated polyhydroxylated amino acids via ester enolate Claisen rearrangement. Synlett.

[75] Kazmaier U., Schneider C. (1998). Application of the asymmetric chelate enolate Claisen rearrangement to the synthesis of unsaturated polyhydroxylated amino acids. Synthesis.

[76] Kazmaier U., Schneider C. (1998). Application of the asymmetric chelate-enolate Claisen rearrangement to the synthesis of 5-epi-isofagomine. Tetrahedron Lett..

[77] Marti C., Carreira E.M. (2005). Total synthesis of (-)-spirotryprostatin B: Synthesis and related studies. J. Am. Chem. Soc..

[78] Li P., Xu J.C. (2000). 1-Ethyl 2-halopyridinium salts, highly efficient coupling reagents for hindered peptide synthesis both in solution and the solid-phase. Tetrahedron.

[79] Li P., Xu J.C. (2000). 2-Bromo-1-ethyl pyridinium tetrafluoroborate (BEP): A powerful coupling reagent for *N*-methylated peptide synthesis. Chem. Lett..

[80] Barbie P., Kazmaier U. (2016). Total synthesis of cyclomarins A, C and D, marine cyclic peptides with interesting anti-tuberculosis and anti-malaria activities. Org. Biomol. Chem..

[81] Junk L., Papadopoulos E., Kazmaier U. (2021). Tryptophan *N*-1-alkylation: Quick and simple access to diversely substituted tryptophans. Synthesis.

[82] Bürstner N., Roggo S., Ostermann N., Blank J., Delmas C., Freuler F., Gerhartz B., Hinniger A., Hoepfner D., Liechty B. (2015). Gift from Nature: Cyclomarin A Kills Mycobacteria and Malaria Parasites by Distinct Modes of Action. ChemBioChem.

[83] Schmitt E.K., Riwanto M., Sambandamurthy V., Roggo S., Miault C., Zwingelstein C., Krastel P., Noble C., Beer D., Rao S.P.S. (2011). The natural product cyclomarin kills *Mycobacterium tuberculosis* by targeting the ClpC1 subunit of the caseinolytic protease. Angew. Chem. Int. Ed..

[84] Vasudevan D., Rao S.P.S., Noble C.G. (2013). Structural basis of mycobacterial inhibition by cyclomarin A. J. Biol. Chem..

[85] Barbie P., Kazmaier U. (2016). Total synthesis of desoxycyclomarin C and the cyclomarazines A and B. Org. Biomol. Chem..

[86] Kiefer A., Bader C.D., Held J., Esser A., Rybniker J., Empting M., Müller R., Kazmaier U. (2019). Synthesis of new cyclomarin derivatives and their biological evaluation towards *Mycobacterium tuberculosis* and *plasmodium falciparum*. Chem. Eur. J..

[87] Choules M.P., Wolf N.M., Lee H., Anderson J.R., Grzelak E.M., Wang Y.H., Ma R., Gao W., McAlpine J.B., Jin Y.Y. (2019). Rufomycin targets ClpC1 proteolysis in *Mycobacterium tuberculosis* and *M. abscessus*. Antimicrob. Agents Chemother..

[88] Nessar R., Cambau E., Reyrat J.M., Murray A., Gicquel B. (2012). *Mycobacterium abscessus*: A new antibiotic nightmare. J. Antimicrob. Chemother..

[89] Kar N.P., Sikriwal D., Rath P., Choudhary R.K., Batra J.K. (2008). *Mycobacterium tuberculosis* ClpC1. FEBS J..

[90] Schmitz K.R., Sauer R.T. (2014). Substrate delivery by the AAA+ ClpX and ClpC1 unfoldases activates the mycobacterial ClpP1P2 peptidase. Mol. Microbiol..

[91] Wolf N.M., Lee H., Choules M.P., Pauli G.F., Phansalkar R., Anderson J.R., Gao W., Ren J.H., Santarsiero B.D., Lee H. (2019). High-resolution structure of CIpC1-rufomycin and ligand binding studies provide a framework to design and optimize anti-tuberculosis leads. ACS Infect. Dis..

[92] Wolf N., Lee H., Choules M., Klein L., Petukhova V., Tufano M., Phansalkar R., Gao W., Santarsiero B., Lee H. (2018). High-resolution structure of Clpc1-Ntd-Rufomycin complex provides three-dimensional framework for optimization of cyclopeptide anti-Tb drug leads. Protein Sci..

[93] Wolf N.M., Lee H., Nam J., Hong J., Duc N.M., Ho N.A., Lee H., Suh J.W., Pauli G.F., Franzblau S.G. (2019). Structures of CIpC1-NTD with potent anti-TB cyclic peptides Rufomycin and Ecumicin: Implications for the mechanism of action and design of therapeutic agents. Acta Cryst. A.

[94] Xie Q., Yang Z.J., Huang X.M., Zhang Z.K., Li J.B., Ju J.H., Zhang H., Ma J.Y. (2019). Ilamycin C induces apoptosis and inhibits migration and invasion in triple-negative breast cancer by suppressing IL-6/STAT3 pathway. J. Hematol. Oncol..

[95] Intaraudom C., Rachtawee P., Suvannakad R., Pittayakhajonwut P. (2011). Antimalarial and antituberculosis substances from *Streptomyces* sp. BCC26924. Tetrahedron.

[96] Weinhäupl K., Brennich M., Kazmaier U., Lelievre J., Ballell L., Goldberg A., Schanda P., Fraga H. (2018). The antibiotic cyclomarin blocks arginine-phosphate-induced millisecond dynamics in the *N*-terminal domain of ClpC1 from *Mycobacterium tuberculosis*. J. Biol. Chem..

[97] Maurer M., Linder D., Franke K.B., Jager J., Taylor G., Gloge F., Gremer S., Le Breton L., Mayer M.P., Weber-Ban E. (2019). Toxic activation of an AAA plus protease by the antibacterial drug cyclomarin A. Cell Chem. Biol..

